# Laparoscopic adrenalectomy: Surgical techniques

**DOI:** 10.4103/0970-1591.44277

**Published:** 2008

**Authors:** Matthew J. Mellon, Amanjot Sethi, Chandru P. Sundaram

**Affiliations:** Department of Urology, Indiana University School of Medicine, Indianapolis, IN 46202, USA

**Keywords:** Adrenalectomy, adrenal gland, endocrine, laparoscopy, surgical technique

## Abstract

Since its first description in 1992, laparoscopic adrenalectomy has become the gold standard for the surgical treatment of most adrenal conditions. The benefits of a minimally invasive approach to adrenal resection such as decreased hospital stay, shorter recovery time and improved patient satisfaction are widely accepted. However, as this procedure becomes more widespread, critical steps of the operation must be maintained to ensure expected outcomes and success. This article reviews the surgical techniques for the laparoscopic adrenalectomy.

## INTRODUCTION

Since Gagner *et al.*, described the first laparoscopic adrenalectomy (LA), this minimally invasive surgical approach has almost replaced the open adrenalectomy in the management of small and medium-sized adrenal lesions.[1] The advantages of LA include shorter hospital stays, decreased postoperative pain, improved recovery times, and better cosmetic results.[2] In addition, difficulty with open surgical exposure and the small size of the adrenal gland make this organ particularly amenable to a minimally invasive technique. The anatomical location of the adrenal gland has led to a number of laparoscopic approaches, including posterior or lateral retroperitoneal, transthoracic, and lateral transperitoneal. In this paper we review the varied surgical techniques used for LA.

## INDICATIONS AND CONTRAINDICATIONS

The most common indication for LA is a unilateral benign adrenal lesion. This includes incidentalomas, pheochromocytomas, aldosteronomas, and Cushing's syndrome. Other less common indications involve adrenal cysts, myelolipomas, androgen-secreting tumors, ganglioneuromas, and adrenal hemorrhage.[3] Bilateral LA has also been used for cases of Cushing's syndrome refractory to medical management and bilateral adrenal neoplasms. In our experience, surgeons should be wary of approaching lesions greater than 8 cm early in their series. Contraindications include tumors larger than 12 cm likely containing malignancy and local tumor invasion into adjacent structures. Debate still remains regarding the utility of LA for metastatic adrenal disease and adrenocortical carcinoma. Relative contraindications include morbid obesity, significant cardiopulmonary disease, coagulopathy, and other conditions not amenable to laparoscopy.

## PREOPERATIVE PREPARATION

We do not routinely perform mechanical bowel preparation prior to LA. Preoperative antibiotic prophylaxis is administered prior to beginning the procedure. Anti-thrombotic stockings are placed in the waiting room prior to induction of anesthesia and a sequential compression device is utilized throughout the case. We generally place an oral-gastric tube and Foley catheter prior to initiation of the procedure and these are removed at its conclusion.

## TRANSPERITONEAL SURGICAL APPROACH

Transperitoneal approach offers the greatest visualization of the operative field, reducing intraoperative injuries and ensuring minimal morbidity. In addition to a monitor tower and gas insufflator set at intra-abdominal pressure of 15 mm Hg, we use 0° and 30° 10-mm laparoscopes. One 12-mm and three 5-mm trocars are generally used. Our surgical instruments include suction aspirator, curved ultrasonic shears, right-angle forceps, bipolar forceps, monopolar Endoshears, 5-mm Hem-o-Lok (Weck) clip-applier, Endopouch specimen bag, and PEER retractor [Table 1]. Pneumoperitoneum is established using a Veress needle technique. The open Hasson method to obtain peumoperitoneum may also be used.

**Table 1:** Instruments for transperitoneal laparoscopic adrenalectomy

10-mm 30° and 0° laparoscopes
5-mm 30° and 0° laparoscopes
One 12-mm and three (left) or four (right) 5-mm non-bladed trocars
5-mm Suction Aspirator (Stryker, Kalamazoo, MI)
Ultrasonic curved shears – Harmonic Scalpel (Ethicon Endosurgery, Cincinnati, OH)
Laparoscopic scissors
5-mm right-angle forceps
Graspers- locking and non-locking (2)
Bipolar forceps (Aesculap or Wolf)
5-mm polymer locking clips and applier (Hem-o-Lok- Weck, NC)
10-mm specimen retrieval bag (Ethicon or US Surgical)
PEER retractor (Jarit, Hawthorne, NY)
Diamond-Flex triangular retractor (Snowden-Pencer, Tucker, GA)
Optional: 5-mm Ligasure laparoscopic forceps (Valleylab, Boulder, CO)
Optional: Carter Thomasson Inlet Closure device (Inlet Medical, Eden Prairie, MN)

## PATIENT POSITIONING

After anesthesia induction, the patient is placed in lateral decubitus position with the affected side elevated around 60°. The patient is placed on a bean bag that helps support the patient in the required position. A soft roll is placed under the contralateral axilla. The arms are secured with padding. The contralateral arm is generally positioned on an arm board, bolstered with pillows or soft padding, and secured with tape. The ipsilateral arm is similarly secured on top of the contralateral arm, but can also be supported by a metal L-shaped support that is secured to the table. We have found that flexing the table is not required for the transperitoneal approach. The patient is securely fastened to the table with a two-inch tape over the lower leg, thigh, pelvis and chest, allowing for maximal table rotation during surgery. Also, the potential of open conversion should be considered during positioning.

### Left adrenalectomy

**Figure 1:**
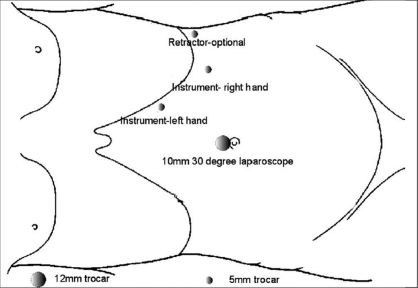
Schematic diagram depicting laparoscopic port placement for left transperitoneal adrenalectomy

**Figure 2:**
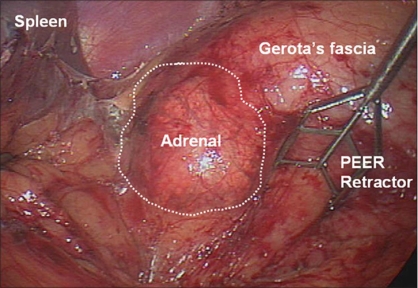
Left adrenalectomy. Exposure of the adrenal gland is obtained by laterally retracting the kidney and adrenal with a PEER retractor

**Figure 3:**
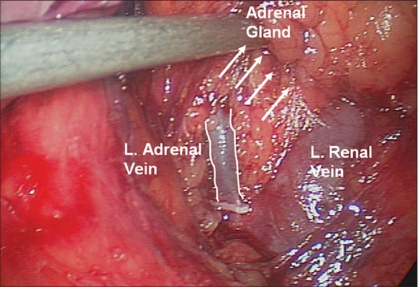
Identification of the left adrenal vein and subsequent division after dissection and clipping

**Figure 4:**
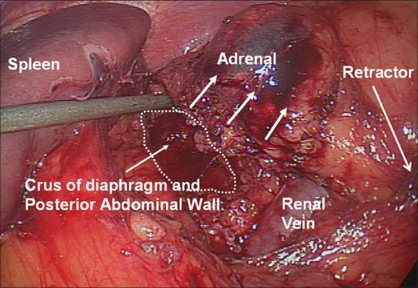
Left adrenalectomy medial dissection. The medial dissection and inferior adrenal dissection is complete, exposing the crus of the diaphragm and the posterior abdominal wall

**Figure 5:**
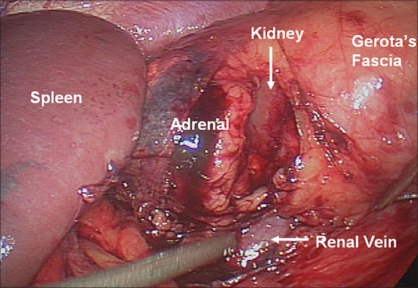
Left adrenalectomy, lateral dissection. The lateral dissection begins with establishing a plane between the upper pole renal cortex and the adrenal gland. It requires careful attention for upper pole renal vessels

**Figure 6:**
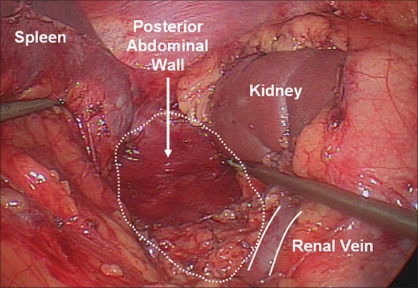
Adrenal bed after adrenalectomy. The adrenal gland with its surrounding periadrenal and peri-renal fat, has been excised

We generally use three 5-mm working ports and one 12-mm camera port for left-sided procedures [Figure 1]. The first 12-mm port is inserted in the umbilicus or at the lateral border of the rectus abdominis muscle just above the level of the umbilicus to accommodate the camera. Two subcostal 5-mm ports are placed; one in the midclavicular line and the other in the lateral border of the rectus abdominis muscle. The third 5-mm subcostal trocar is inserted in the anterior axillary line. Avoid placing these ports too closely together. Doing so can severely restrict freedom of movement and operating space; 8-10 cm apart should be the goal. Obese patients may require placing these ports more laterally. The initiation of the left adrenalectomy requires incising along the white line of Toldt from the splenic flexure to the sigmoid junction to allow mobilization of the left colon. The descending colon is reflected medially and subsequent division of the phrenocolic and splenorenal ligaments allows the colon to fall away out of the visual field [Figure 2]. Dissection is carried out between the Gerota's fascia and the mesocolon. Dissection in this plane will facilitate access to the renal and adrenal vein. The lateral fourth trocar is used for the PEER retractor attached to a self-retaining Endoholder. The retractor is used to retract the kidney laterally during the adrenal dissection. In each case, correct identification of the vasculature must be confirmed prior to isolation and clipping of the adrenal vein [Figure 3]. If the renal vein is not readily identified, the gonadal vein may be located below the renal hilum and followed up to the renal vein. The adrenal vein insertion into the renal vein is medial to the gonadal's termination. We avoid dissecting the adrenal gland prior to vascular ligation, especially in pheochromocytoma cases, to avoid systemic release of catecholamines. The inferior dissection is performed after the adrenal vein has been controlled. Bipolar diathermy is adequate to control the left adrenal vein before it is divided by endoshears or the ultrasonic shears. The 5-mm Hem-o-Lok clips can also be used. Dissection is carried out just superior to the renal vein until the post abdominal wall is identified. The Harmonic Scalpel (Ethicon Endosurgery, Cincinnati, OH) is usually adequate to control small vessels encountered during the inferior and medial adrenal dissection. However, if larger vessels are encountered, bipolar coagulation may be required. The 5-mm Ligasure bipolar device can also be used instead of the Harmonic Scalpel. The muscles of the posterior abdominal wall and the crus of the diaphragm are visualized during medial and inferior adrenal dissection. Lateral retraction of the adrenal and kidney can facilitate this dissection [Figure 4]. Hilar lymphatics of the kidney are avoided unless adrenocortical carcinoma is suspected. Care is taken not to injure branches of the renal artery. Attempts are made to leave periadrenal fat surrounding the gland, to ensure a complete and wide excision of the adrenal gland. This technique allows the surgeon to manipulate the tissue without directly grasping the adrenal, avoiding annoying bleeding. Once the inferior dissection is complete the medial dissection is carried out from the renal vein in a cephalad direction. The medial border for this dissection is the crus of the diaphragm overlying the suprarenal aorta. The lateral dissection is in a plane extending between the renal cortex and the perinephric fat adjacent to the adrenal gland [Figure 5]. This region is usually avascular, but care must be taken to avoid upper pole renal vessels. The superior dissection is the last step before the adrenal gland is free within the abdomen along with periadrenal fat. The left inferior phrenic vein is generally present during the superior dissection and must be carefully identified and divided to avoid troublesome bleeding. The dissected gland is placed in an Endopouch that is inserted through the camera port [Figure 6]. A 5-mm laparoscope is used during this step. Lateral extension of the 10-mm incision will be required to extract the specimen intact. The incision is closed with 0 Polydioxanone suture. If the tumor is large, a separate lower midline or Pfannensteil incision may be used for specimen extraction.

### Right adrenalectomy

**Figure 7:**
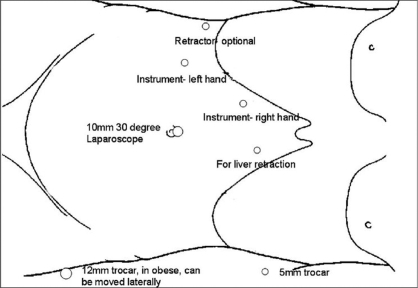
Schematic diagram depicting laparoscopic port placement for right transperitoneal adrenalectomy

**Figure 8:**
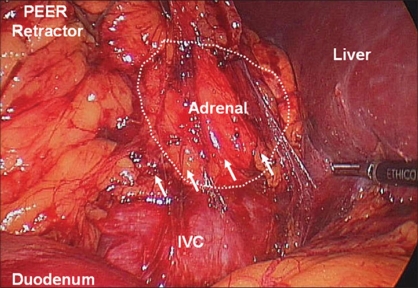
Right adrenalectomy, initial dissection. After duodenal mobilization and incision of the peritoneum to expose the IVC, dissection is carried inferiorly along the IVC just superior to the right renal vein (arrows)

**Figure 9:**
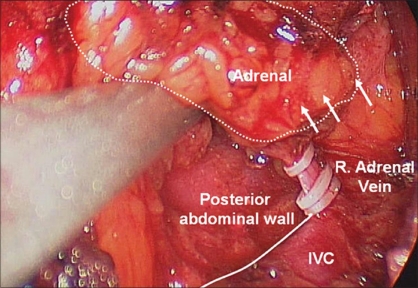
Right adrenalectomy, medial dissection. Continuing cephalad along the IVC, the short right adrenal vein is encountered and divided between clips

**Figure 10:**
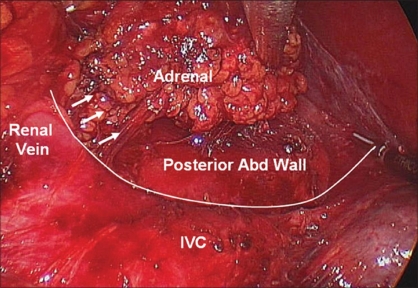
Right adrenalectomy, Completion of medial dissection. The posterior abdominal wall lateral to the IVC is exposed

Five ports are used for right-sided procedures [Figure 7]. The trocars are placed in a mirror image to the left side with the addition of a 5mm in the epigastrium for liver retraction. The epigastric trocar is inserted just to the left of the midline in relation to the lower edge of the liver, so that it does not interfere with the right-handed instrument and trocar. Occasionally, the liver is enlarged and floppy and the grasper may not be adequate for optimal retraction. The Diamond-Flex triangular retractor (Snowden-Pencer, Tucker, GA) inserted through the medial or lateral trocars may then be used for liver retraction. The first step is division of the triangular ligament and careful cephalad retraction of the liver using a locking grasper that is inserted through the epigastric trocar. The grasper holds a fold of the peritoneum or the diaphragm on the lateral abdominal wall, and retracts the liver. Unlike the left side, the colon rarely requires significant mobilization. At this point, the subhepatic inferior vena cava (IVC) should be identified, lateral and posterior to the gall bladder. This phase can be difficult in obese patients or those with Cushing's syndrome where excess adipose tissue is present. The peritoneum along the lateral aspect of the IVC is incised to expose the IVC just below its intrahepatic course. The duodenum often needs to be mobilized to expose the IVC. Dissection is next carried inferiorly by incising the peritoneum along the lateral edge of the vena cava to the superior edge of the renal vein [Figure 8]. Further dissection extends posteriorly, bordering the superior aspect of the renal vein, for the inferior adrenal dissection. The posterior abdominal wall is encountered, ensuring that the renal artery or its branches are safeguarded. The inferior adrenal gland and periadrenal tissue is retracted laterally with the PEER retractor, as the medial dissection is performed in a cephalad direction. The dissection proceeds superiorly just lateral to the cava until the short right adrenal vein is identified. Once encountered, this vessel is divided between double 5-mm locking polymer clips [Figure 9]. As the dissection continues superiorly, small branches of the inferior phrenic vessels may be encountered and are cauterized with bipolar instruments. Dissection of the gland is subsequently carried out with care taken at the medial aspect where the wall of the IVC closely approximates the adrenal. The adrenal may extend posterior to the cava and this dissection is completed by following the wall of the cava and gentle medial retraction of the IVC [Figure 10]. Once the medial dissection is completed, the lateral dissection is performed just as described for the left adrenalectomy. Once complete mobilization is accomplished and hemostasis is achieved, the specimen is removed with the EndoPouch.

## LATERAL RETROPERITONEAL SURGICAL APPROACH

The retroperitoneal approach has the advantage of avoiding the intra-abdominal organs and allowing direct access to the adrenal. This technique does not provide the same operative exposure as transperitoneal attempts and tumors larger than 7 cm may not be amenable to this technique. Additionally, there is a general lack of anatomical landmarks, making dissection more challenging. The major indication for the retroperitoneal approach is previous intra-abdominal surgery with the presence of adhesions. Our institution has adopted a technique similar to the method described by Sung *et al.*[4]

### Patient positioning

Patients are placed in a lateral decubitus position. To fully expand the operating space between the 12^th^ rib and the iliac crest, the table is flexed. The goal is to expand the distance between the costal margin and the iliac crest. All extremities are meticulously padded and secured as described for transperitoneal adrenalectomies above to avoid neuromuscular injuries. Surgeons must be cautious when positioning in the full flank position for prolonged periods of time.

### Retroperitoneal access

Most surgeons use the open Hasson technique to gain access to the retroperitoneum. The 2 cm skin incision is made approximately 2 cm below the inferior edge of the 12^th^ rib. The underlying muscle layers are bluntly separated and the retroperitoneum entered by dividing the thoracolumbar fascial layer with a hemostat. With careful finger dissection, a potential space is created below the fascia to allow placement of the balloon dilator (Origin Medsystems, Menlo Park, CA). Care should be taken to avoid puncturing Gerota's fascia during this step. Approximately 800 mL of air is inflated into the balloon to create the working space. The device is subsequently deflated and advanced cephalad along the psoas muscle in the retroperioteum towards the level of the diaphragm. The balloon is expanded and deflated a second time and then removed. It can be helpful to place a laparoscope concurrently in the working cavity to aid with the dilation process. A 10-mm trocar is inserted and pneumoperitoneum established. Two additional 5-mm ports are used. One trocar is placed in the anterior axillary line midway between the costal margin and the iliac crest. The third port is located posteriorly between the 12^th^ rib and the iliac crest along the lateral border of the sacrospinalis muscle. A fourth port (5-mm) is inserted for retraction of the kidney and is placed cephalad to the first port in the anterior axillary line. The psoas muscle posteriorly is an important landmark to ensure that the kidney and adrenal gland are located anteriorly.

### Left adrenalectomy

After port placement and balloon creation of the operating space, we incise Gerota's fascia posterior to the upper renal pole with the Harmonic Scalpel. By extending this dissection around the superior aspect of the kidney, the unmobilized adrenal will remain stationary, allowing the kidney to fall away from the gland. The use of a 30° laparoscope greatly aids this maneuver. The surgeon must be aware at this juncture of accessory renal vessels which can be injured during this step. Next, isolation of the main renal vessels is performed. Care must be taken to avoid dissecting too far caudally into the renal hilum. Continuing the dissection medially along the renal vein, the main adrenal vein is encountered. After correct identification, this vessel is then divided between clips. As the mobilization extends over the superior aspect of the gland, small inferior phrenic vessels are controlled with the Harmonic Scalpel or bipolar cautery. Finally dissection is completed along the infero-medial aspect of the adrenal. Occasionally, if the main adrenal vein has not been identified at this point, it is usually found along this aspect of the gland. Using a 5-mm laparoscope for visualization, an EndoPouch retrieval device is inserted into the primary port and the specimen is removed.

### Right adrenalectomy

Ports are placed in a similar mirror image fashion to the left. Again the investing fascial layer around the right kidney is opened transversely along the upper renal pole and circumferential dissection continued, creating a potential space between the adrenal and the kidney. At this point the IVC is identified and dissection is extended superiorly along the lateral edge of the cava. The right adrenal vein is likely encountered at this point, branching off the medial aspect of the gland. This vessel is again divided between clips. The mobilization of the right adrenal is completed in similar fashion to the left with care given towards recognizing the inferior phrenic vessels while dissecting along the inferior aspect of the diaphragm. Rarely, a fan retractor is used to medially displace the liver to allow proper visualization and working space.

## POSTERIOR RETROPERITONEAL SURGICAL APPROACH

Though most retroperitoneoscopic approaches to the adrenal gland are performed via a lateral flank technique, as described above, this approach attempts to dissect the gland from the superior pole of the kidney prior to adrenal vessel ligation. A retroperitoneal posterior lumbar approach allows direct access to the adrenal vessels without invading the peritoneal space. This technique was initially reported by Walz, *et al.*, but it is not commonly employed for adrenalectomies today.[5]

## TRANSTHORACIC SURGICAL APPROACH

For a very select group of patients where prior surgical attempts in the transabdominal and retroperitoneal cavities precludes a laparoscopic approach, a minimally invasive approach to the adrenal gland has been suggested. Gill *et al.*, first introduced the technique of the thoracoscopic transdiaphragmatic adrenalectomy.[6] In carefully selected patients, this technique appears to be an effective alternative, though few reports of its use in the literature exist.

## LAPAROSCOPIC BILATERAL ADRENALECTOMY

Bilateral adrenalectomy is performed for bilateral adrenal hyperplasia associated with Cushing's syndrome that is refractory to medical management. The surgery may also be required for bilateral adrenal neoplasms. Patients demonstrating an excess of cortisol have increased adipose tissue and some have argued that a laparoscopic approach in these patients is most appropriate.[7] In addition, patients with Cushing's syndrome have an increased rate of morbidity due to poor wound healing and increased risk of thromboembolic events. Prior to surgical intervention care must be taken to medically optimize these individuals. Though each of the above-described techniques may be employed, we generally utilize a lateral transperitoneal approach. The larger tumor or the more difficult side should be performed first. After one side is completed, the patient is re-positioned and re-draped for the opposite side.

## LAPAROSCOPIC PARTIAL ADRENALECTOMY

Since Jeschke *et al.*, demonstrated the safety and efficacy of laparoscopic partial adrenalectomy for aldosteronomas, the laparoscopic approach has increasingly been employed for adrenal-sparing procedures.[8] With the goal of preserving adrenal function in patients with adrenal lesions, the laparoscopic approach offers improved visualization and ability to distinguish normal parenchyma from tumor. The initial operative technique follows closely the procedures described previously. After insufflation and careful exposure of the adrenal gland, we attempt to excise a thin rim of normal adrenal tissue surrounding the adrenal nodule, using the Harmonic Scalpel. The adrenal vein is routinely left intact unless tumor location necessitates bipolar coagulation. It is important to minimize dissection adrenal's connective tissueto preserve vascular supply to the remaining gland. The importance of successfully identifying the limits of the adrenal neoplasm prior to initiating dissection cannot be stressed enough. Intraoperative ultrasound using a 10-mm laparoscopic steerable flexible probe is helpful in delineating the extent of the tumor. Bipolar coagulation and Fibrin glue or Floseal (Baxter) is used for assistance with hemostasis after the partial adrenalectomy.

## SURGICAL COMPLICATIONS

Small liver injuries can occur during retraction or adrenal dissection on the right. Bipolar coagulation, Endo-peanut compression, or placement of Surgicel® generally leads to hemostasis. Additionally, the argon beam can be useful for troublesome bleeding. Rarely do injuries lead to open conversion. Vascular injuries, especially vena cava trauma, comprise almost 7% of all LA complications and are the leading cause for conversion.[9] Small caval injuries <2mm may be compressed and coagulant agents applied with good success. Increasing the intra-abdominal pressure to 20 mm Hg could help with the bleeding. If bleeding continues, laparoscopic suturing is required, occasionally with placement of an additional trocar. This technique should only be attempted by experienced laparoscopic surgeons. Pleural injuries and pneumothoraces occasionally occur, requiring diaphragm suture closure without a chest tube. Postoperatively if the pneumothorax is significant, a chest tube may be required. Injury to the spleen and pancreas on the left and the duodenum and liver on the right must be avoided.

## CONCLUSION

Laparoscopic adrenalectomy is a safe and effective technique for the surgical removal of adrenal masses. This minimally invasive approach provides clear advantages over open resection. With careful patient selection and careful surgical technique, successful outcomes should be expected.
